# Editorial: Diabetic renal tubulointerstitial disease

**DOI:** 10.3389/fendo.2023.1303514

**Published:** 2023-11-01

**Authors:** Katsumi Iizuka, Kanako Deguchi

**Affiliations:** ^1^ Department of Clinical Nutrition, Graduate School of Medicine, Fujita Health University, Toyoake, Japan; ^2^ Food and Nutrition Service Department, Fujita Health University Hospital, Toyoake, Japan

**Keywords:** diabetic kidney disease, renal tubulointerstitial disease, diabetes mellitus, GWAS, cellular senescence

Diabetic kidney disease (DKD) is one of the three microvascular complications of diabetes. DKD remains the leading cause of end-stage kidney disease (ESRD) in many countries, and epidemiological studies suggest that approximately 40% of patients with diabetes will develop it (1). DKD involves classical diabetic nephropathy and tubulointerstitial inflammation and fibrosis. Progressive albuminuria was previously deemed the main factor in the development of diabetic nephropathy. However, it is now known that diabetic nephropathy can progress even without early trace albuminuria (2). The renal tubules and interstitium involve renal tubules (proximal, distal, and collecting), dendritic cells, macrophages, lymphocytes, lymphatic endothelial cells, renin or erythropoietin-producing cells, and fibroblasts, respectively. These suggest that renal tubule-interstitial regions are essential in regulating water-electrolytes, the immune system, and the endocrine system (3). Therefore, it is not hard to imagine that this inflammation and fibrosis in the tubulointerstitium can lead to reduced renal function and renal failure. The tubulointerstitial damage is correlated with the deterioration of renal function. In patients with diabetes, increased renal interstitial fibrosis directly correlates with reduced renal function, such as creatinine clearance (4). Moreover, recent studies have emphasized the crucial role of tubulointerstitial injury as a mediator of the progression of kidney disease (5, 6). As the topic of this editorial, we focused on diabetic renal tubulointerstitial disease.

Wang et al. reviewed the molecular mechanism of tubular injury in diabetic kidney disease. The authors state that several factors, such as hyperglycemia, lipid accumulation, oxidative stress, hypoxia, the renin-angiotensin-aldosterone system (RAAS), endoplasmic reticulum stress, inflammation, epithelial–mesenchymal transition, and programmed cell death (including apoptosis, autophagy, pyroptosis, and ferroptosis), lead to renal tubular injury and exacerbate DKD (Wang et al.). Moreover, the authors summarized potential treatments against the progression of tubular damage. They described the role of drugs such as sodium-glucose cotransporter-2 inhibitor (SGLT2i), glucagon-like peptide 1Receptor Agonists (GLP-1RA), Renin-angiotensin-system inhibitors (RASi), mineralocorticoid receptor antagonists, and stem cell therapies. In our daily clinical practice, we experienced a case of rapidly declining estimated glomerular filtration rate and tubular intestinal injury confirmed by renal biopsy, which was improved by the combination of SGLT2i, GLP-1RA, and RASi, and we realized the effectiveness of the drugs (7). Stem cells migrate to the injured sites and repair them through directional differentiation, paracrine effects, and modulation of the immune response (8). Stem cell-based cell therapy is expected to improve renal function effectively [Wang et al.]. It improves proteinuria, fibrosis, inflammation, apoptosis, epithelial-mesenchymal transition, and oxidative stress. However, it is unclear to what stage of disease this treatment should start. Perhaps it would be effective only when fibrosis is not sufficiently advanced. In the future, a marker to clinically quantify tubular damage and fibrosis and diagnose it early. Xu et al. also published the review Advances in Understanding and Treating Diabetic Kidney Disease: Focus on Tubulointerstitial Inflammation Mechanisms. The author’s review was focused on inflammatory mechanisms in the tubulointerstitium.

Luo et al. reported cellular senescence-associated signatures in diabetic kidney disease. The authors performed the Gene Set Enrichment Analysis (GSEA) algorithm to evaluate the activity of senescence pathways in DKD patients. Furthermore, the authors identified module genes related to cellular senescence pathways through the weighted gene coexpression network analysis algorithm. They constructed a cellular senescence-related signature (SRS) risk based on five hub genes (LIMA1 (LIM domain and actin binding 1), ZFP36 (ZFP36 ring finger protein), FOS (FOS proto-oncogene), IGFBP6 (insulin-like growth factor binding protein 6), and CKB (creatine kinase B)). The patients with the high SRS risk scores had lower GFR and higher expression of fibrotic genes than those with the low SRS risk scores. Moreover, the patients with high SRS risk scores exhibited extensive inhibition of mitochondrial functions (oxidative phosphorylation and fatty acid metabolism) and increased immune cell infiltration. The proximal tubules are essential for mitochondrial function because they require ATP during glucose and sodium reabsorption from urine (9). Furthermore, mitochondrial defects in renal proximal tubules contribute to increased oxidative stress and activation of inflammatory pathways, thereby causing progressive kidney function decline and fibrosis. Thus, proximal tubule changes strongly correlate with the glomerular filtration rate. These findings suggest the usefulness of the SRS risk score. These results reconfirmed the essential roles of cellular senescence in the progression of DKD. However, some questions remain. The paper does not mention the expression levels of LIMA1, ZFP36, FOS, IGFBP6, and CKB in the kidney, especially in the renal tubules. Explaining the role of the kidney concerning each molecule will benefit the readers. In addition, as risk assessment for gene expression requires kidney biopsy, quantitative surrogate marker criteria in blood or urine will be needed. We await further research in this regard.

There has been recently significant progress in genetic and epigenetic research. In this respect, not all papers dealt with renal tubulointerstitial injury. Some authors reported that the genotype of albuminuria in DKD appears to be different from that of eGFR (1). The proteinuria phenotype seems to cluster with genes expressed by podocytes (1). In contrast, the genotype of eGFR is very similar in those with and without diabetes (1). Associations have also been reported between hyperglycemia-induced DNA methylation and albuminuria, glycemic control, baseline eGFR, and eGFR decline (1). Moreover, genome-wide association and quantitative traits (GWAS, meQTL, and eQTL) revealed the critical roles of proximal tubules and metabolism in kidney function regulation (10). The study also showed the causal role of SLC47A1 in kidney disease. Thus, considering genetic factors, gene expression, and epigenetic factors may contribute to understanding the etiology of diabetic kidney disease in humans.

## Author contributions

KI: Writing – original draft, Writing – review & editing. KD: Writing – original draft.
